# High risk of dengue type 2 outbreak in French Polynesia, 2017

**DOI:** 10.2807/1560-7917.ES.2017.22.14.30505

**Published:** 2017-04-06

**Authors:** Maite Aubry, Yoann Teissier, Mihiau Mapotoeke, Anita Teissier, Marine Giard, Didier Musso, Van-Mai Cao-Lormeau

**Affiliations:** 1 Unit of Emerging Infectious Diseases, Institut Louis Malardé, Papeete, Tahiti, French Polynesia; 2 ED 474, Université Sorbonne Paris Cité, Paris, France; 3 Direction de la Santé de la Polynésie française, Papeete, Tahiti, French Polynesia

**Keywords:** Arbovirus, Dengue virus, Serotype 2, outbreak, French Polynesia, Pacific

## Abstract

In French Polynesia, the four serotypes of dengue virus (DENV-1 to -4) have caused 14 epidemics since the mid-1940s. From the end of 2016, an increasing number of Pacific Island Countries and Territories have reported DENV-2 outbreaks and in February 2017, DENV-2 infection was detected in French Polynesia in three travellers from Vanuatu. As DENV-2 has not been circulating in French Polynesia since December 2000, there is high risk for an outbreak to occur.

In February 2017, three travellers from Vanuatu were diagnosed with dengue virus serotype 2 (DENV-2) infection in French Polynesia (a French collectivity in the South Pacific). As DENV-2 has not been circulating in the country for ca 16 years, we discuss here the risk factors that could contribute in a near future to the re-emergence of this virus in French Polynesia and to subsequent dissemination to other, not yet affected, Pacific islands and continental countries having close links with European overseas countries and territories.

## Detection of imported cases of DENV-2 infections in French Polynesia

**Figure 1:**
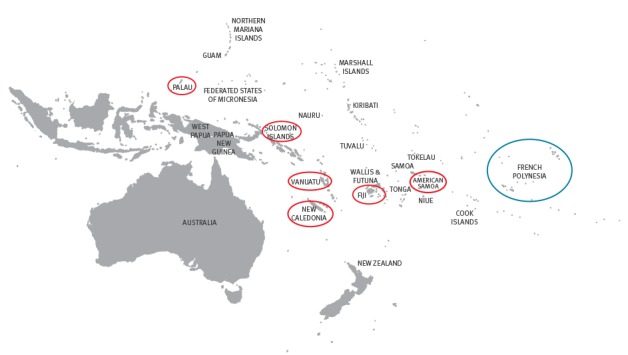
Map of DENV-2 epidemics reported in the Pacific Island Countries and Territories, February–March 2017

A soccer contest involving participants from Fiji, New Caledonia, New Zealand, Papua New Guinea, Samoa, Solomon Islands and Vanuatu was organised in French Polynesia in February 2017. Because of the ongoing circulation of DENV-2 in several of these Pacific Island Countries and Territories (PICTs) (Figure 1) [1,2], surveillance measures were strengthened by the French Polynesia Direction of Health. 

DENV: dengue virus.

Areas where DENV-2 epidemics have been recorded are surrounded by red circles. French Polynesia is indicated by a blue circle.

Source: [1,2]; map downloaded from: https://www.shutterstock.com/g/Armita.

Participants who declared febrile illness after their arrival in French Polynesia were immediately examined by a medical practitioner, and a blood sample was collected and sent to the Institut Louis Malardé (Papeete, Tahiti, French Polynesia) for DENV diagnosis and DENV genotyping by real-time RT-PCR, using previously published oligonucleotide primers and probe [3]. Three serum samples received from participants from Vanuatu tested positive for DENV-2. Two additional serum samples collected from participants from Vanuatu, and four serum samples collected from participants from Papua New Guinea, tested negative for all four serotypes of DENV.

## Phylogenetic analysis

The complete envelope gene of the DENV-2 strains isolated from the three participants was sequenced with the Abi 3500 genetic analyzer (Applied Biosystems, US), using primers D2/618V (5’-ACCAGAAGACATAGATTGTTGGTGC-3’), DEN-2F (5’- CAGGTTATGGCACTGTCACGA-3’), DEN-2C (5’-CCATCTGCAGCAACACCATCT-3’), D2RS2271 (5’-CCCATAGATTGCTCCGAAAAC-3’) and D2/2578 (5’-TTACTGAGCGGATTCCACAGATGCC-3’).

**Figure 2:**
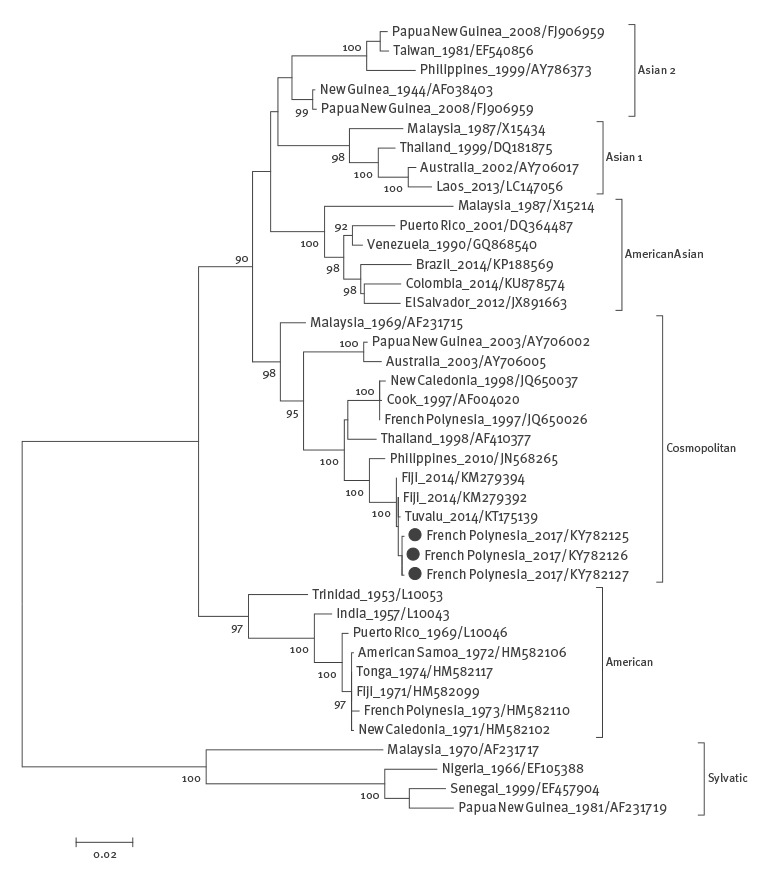
Phylogenetic analysis of DENV-2 strains isolated in French Polynesia, February 2017 (n = 3)

Phylogenetic analysis showed that the three DENV-2 strains imported to French Polynesia from Vanuatu (GenBank accession numbers: KY782125, KY782126 and KY782127) belonged to the Cosmopolitan genotype, and were closely related to strains collected in 2014 in Tuvalu and Fiji, with percentages of homology of more than 99.7% (Figure 2).

DENV: dengue virus.

The evolutionary history was inferred by using the maximum likelihood method based on the Kimura 2-parameter model [24]. The percentage of trees in which the associated taxa clustered together is shown for values higher than 90 next to the branches (1,000 replicates). Evolutionary analyses were conducted in MEGA7 [25]. Each strain is labelled by country _ date of origin / GenBank accession number. The DENV-2 strains isolated in French Polynesia in February 2017 are marked with a black circle.

## Background

**Figure 3:**
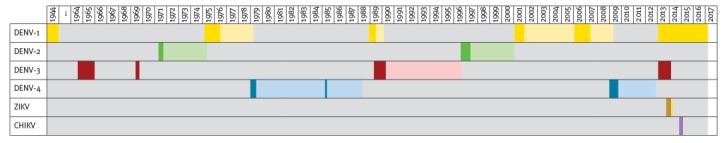
The circulation of the four dengue virus serotypes and of Zika and chikungunya viruses in French Polynesia, 1944–2017

CHIKV: chikungunya virus; DENV: dengue virus; ZIKV: Zika virus.

In French Polynesia, a French Overseas collectivity of ca 270,000 inhabitants in the south-east Pacific, the four serotypes of DENV have caused successive epidemics since the 1940s, and outbreaks due to Zika (ZIKV) and chikungunya (CHIKV) viruses have also been reported recently [4-10]. The epidemiology of DENV in French Polynesia, as in several other PICTs, is characterised by the long-term predominance of a single serotype; its transmission can persist in an endemic way during 4–5 years until the virus causes a new outbreak or is replaced by another serotype [6,7,11,12]. In contrast to DENV serotypes 1, 3 and 4 that have caused several epidemics during the past 16 years (DENV-1 in 2001, 2006–07 and 2013–17; DENV-3 in 2013–14; and DENV-4 in 2009) (Figure 3) [4-10,13], the last DENV-2 outbreak occurred in 1996–97 [11] and the last report of autochthonous DENV-2 infection was in December 2000.

Bright colours indicate epidemic periods and pale colours indicate inter-epidemic periods with presence of circulating virus.

Epidemic periods in French Polynesia are defined by the detection of a minimum of 10 positive dengue cases per week during at least two consecutive weeks.

Source [4-10,13];

## Discussion

Previous epidemiological studies conducted in French Polynesia showed that the sustained transmission of a predominant DENV serotype follows a periodic cycle of 12 years for DENV-1 and ca 20 years for the three other serotypes. Because the islands’ population is small and there is little migration, it has been suggested that this time period is necessary to renew the proportion of non-immune hosts [7]. The absence of DENV-2 circulation during the past 16 years, together with the results of a serosurvey conducted in blood donors in 2011–13 that showed a lower level of herd immunity against DENV-2 than the other DENV serotypes [14], highlight the risk for a large DENV-2 outbreak in French Polynesia.

DENV-2 is currently circulating in several PICTs including American Samoa, Fiji, New Caledonia, Palau, Solomon Islands and Vanuatu. Several DENV outbreaks in French Polynesia resulted from the importation of viral strains from other PICTs, e.g. DENV-4 in 2009 [6] and DENV-1 in 2013 [15]. Frequent tourist exchanges and sporting, cultural and religious events organised between the PICTs increase the risk of virus introduction into French Polynesia, as illustrated by the detection of DENV-2 infection in three travellers coming from Vanuatu to participate in a soccer contest. Phylogenetic analysis confirmed that the DENV-2 strains isolated from these participants belonged to the same lineage as viral strains isolated in other PICTs (Tuvalu and Fiji) in 2014. Although no subsequent autochthonous DENV-2 infections have been detected so far, the occurrence of an outbreak in the coming weeks or months cannot be excluded. In January 2009, two imported DENV-4 infections were detected in inhabitants of French Polynesia returning from New Caledonia where an epidemic had just started [6]. Despite increased surveillance by the French Polynesia Direction of Health and the reinforcement of vector control measures by the Public Health and Hygiene Department, a DENV-4 outbreak was declared 2 months later.

Due to the combination of risk factors exposed above, the occurrence of a new DENV-2 outbreak is to be expected in French Polynesia. Strengthened surveillance measures apply to travellers arriving from countries where DENV-2 is circulating: serotype-specific diagnosis is requested for any suspicion of DENV infection; travellers arriving from New Caledonia, Cook Islands and New Zealand (the transit hub for most PICTs) are informed at arrival about the risk of DENV-2 importation into French Polynesia (awareness-raising flyers, television spots and posters).

Populations of the PICTs have suffered severely from outbreaks of arthropod-borne virus (arbovirus) infections during the past 3 years [10,16,17]. Surveillance is a key factor to anticipate and possibly prevent the spread of arboviruses between the PICTs. Effective surveillance requires timely and reliable data sharing on arbovirus circulation in the region; these data are therefore made available and frequently updated by the Pacific Public Health Surveillance Network (https://www.pphsn.net/). Such information should also be of international interest. Indeed, as recently illustrated with ZIKV, large outbreaks caused by emerging arboviruses in the PICTs can result in virus importation and further autochthonous transmission in non-endemic countries, e.g. in Europe and the Americas [17,18]. Autochthonous transmission of DENV in Europe and North America has already been reported [19-23]. The occurrence of a DENV-2 outbreak in the coming months in French Polynesia would increase the risk of virus importation into such non-endemic countries, particularly mainland France, during the most favourable season for vector-borne transmission.

## Erratum

The authors first and last names were originally published in the wrong order. This was corrected on 7 April 2017. We apologise for the mistake.
