# Erratum for Euro Surveill. 2017;22(14)

**DOI:** 10.2807/1560-7917.ES.2017.22.15.30507

**Published:** 2017-04-13

**Authors:** Eurosurveillance editorial team

**Affiliations:** 1 European Centre for Disease Prevention and Control (ECDC), Stockholm, Sweden

In the article ‘High risk of dengue type 2 outbreak in French Polynesia, 2017’ by Aubry et al., published on 6 April 2017, the authors first and last names were originally published in the wrong order. This was corrected on 7 April 2017. We apologise for the mistake.

